# Guidelines for Telemetry Studies on Snow Leopards

**DOI:** 10.3390/ani12131663

**Published:** 2022-06-28

**Authors:** Örjan Johansson, Shannon Kachel, Byron Weckworth

**Affiliations:** 1 Grimsö Wildlife Research Station, Swedish University of Agricultural Sciences, 73993 Riddarhyttan, Sweden; 2 Snow Leopard Trust, 4649 Sunnyside Avenue North, Seattle, WA 98103, USA; 3 Panthera, 8 West 40th Street, 18th Floor, New York, NY 10018, USA; skachel@panthera.org (S.K.); bweckworth@panthera.org (B.W.)

**Keywords:** animal welfare, capture, collar, felid, GPS, immobilization, *Panthera uncia*, trapping

## Abstract

**Simple Summary:**

Satellite collars and other tracking (telemetry) devices fitted on wild animals can provide insights into species’ habitat requirements, movements, space and resource use, thermoregulation, life history, and demographics. Such information is particularly important for the conservation and management of elusive and threatened species like the snow leopard (*Panthera uncia*). Deploying tracking devices, however, exposes targeted and non-targeted animals to non-trivial risks—for example, trapping-induced injuries or death, adverse reactions to immobilizing drugs, and physiological and behavioral impacts from inappropriately-sized collars. The implicit ethical considerations and tradeoffs are not always obvious, and are often underappreciated by researchers and managers responsible for conducting or approving proposed telemetry projects. Here, we aim to (1) help researchers, managers, and permitting agencies better understand if and when the risks inherent in telemetry studies are ethically justifiable; and (2) help researchers develop efficient and ethical procedures for planning telemetry studies and associated capturing and handling protocols for snow leopards. Telemetry studies that are undertaken with clearly-articulated purpose, well-vetted and comprehensive protocols, and a sustained commitment of resources can play a vital role in conservation.

**Abstract:**

Animal-borne tracking devices have generated a wealth of new knowledge, allowing us to better understand, manage and conserve species. Fitting such tracking devices requires that animals are captured and often chemically immobilized. Such procedures cause stress and involve the risk of injuries and loss of life even in healthy individuals. For telemetry studies to be justifiable, it is vital that capture operations are planned and executed in an efficient and ethical way. Project objectives must be clearly articulated to address well-defined knowledge gaps, and studies designed to maximize the probability of achieving those goals. We provide guidelines for how to plan, design, and implement telemetry studies with a special emphasis on snow leopards that are typically captured using foot snares. We also describe the necessary steps to ensure that captures are conducted safely, and with minimal stress to animals.

## 1. Introduction

For wildlife management and conservation programs to be impactful and cost-effective, foundational knowledge of the target species’ biology is essential. To collect the information needed, it is often necessary to study some number of individual animals using tracking (telemetry) devices, especially for secretive species that are otherwise difficult to observe, such as many felids [1,2]. Telemetry studies can provide valuable data relevant to species biology, including movement dynamics, resource selection, foraging behavior, and demographic processes like cause-specific mortality, fecundity, and dispersal, e.g., [1,3,4,5,6,7]. Fitting tracking devices—typically collars in the case of terrestrial carnivores—requires that animals are captured and immobilized. To safeguard animal welfare, and to ensure that data are representative of non-sampled individuals, it is vital that the transmitters affect individuals minimally and that capture protocols meet high standards of animal welfare [8,9]. A telemetry study can fail, or produce biased results, if the transmitters affect the behavior, reproduction, or survival of tagged individuals [9,10]. Both physical capture and chemical immobilization involve stress and the risk of injuries or mortality for the captured animals [11,12]. Consequently, telemetry studies should only be considered if there is a realistic probability of achieving the stated research, conservation, or management goals. Poorly planned and executed capture programs can undermine those goals, compromise animal safety and welfare, jeopardize research and conservation outcomes, and damage perceptions of wildlife management and conservation efforts amongst the public [13,14].

**Figure 1:**
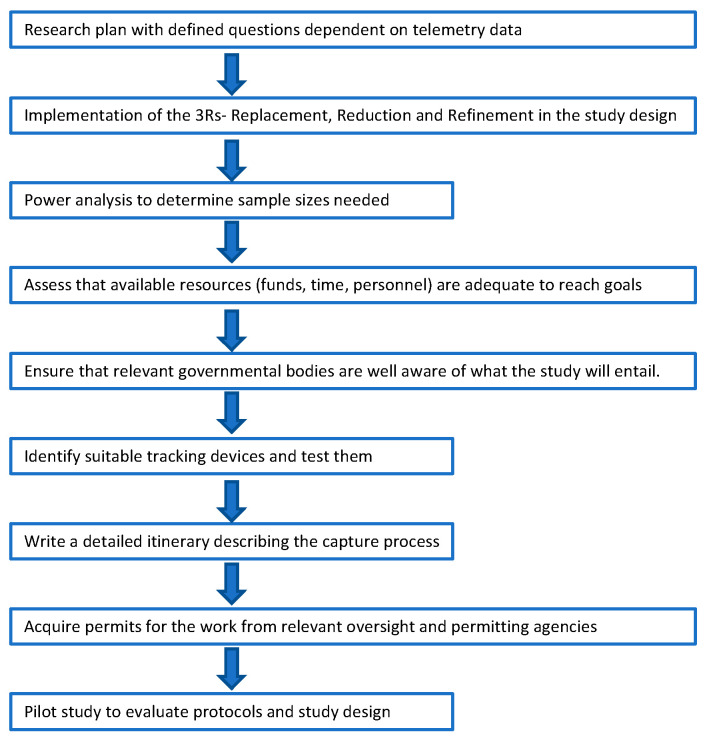
Flowchart summarizing the main steps needed to launch a telemetry study.

Here, we provide guidelines intended to help researchers, wildlife agencies, and conservation organizations evaluate, design, and implement plans for collaring studies and to determine whether a proposed study is sufficiently justified, given the risks to the animals involved. The veterinary procedures and considerations involved in such studies, while being critically important, lie outside the purview of our article. We nevertheless underscore that any effort to capture and handle live animals must involve direct collaborations with qualified veterinarians to develop and practice safe and ethical capture and immobilization protocols. While our guidelines are written with an emphasis on snow leopards, our recommendations for designing and evaluating collaring studies have general applicability across species, for a summary see Figure 1.

## 2. Evaluation

To determine the validity of any proposed collaring study, the initial step is to formulate a clear research plan, outlining and justifying each question that the study seeks to answer, and articulating if and how telemetry data are necessary for addressing those questions. Are the study goals (a) exploratory—i.e., describing patterns without reference to explicit *a priori* hypotheses; (b) inferential—i.e., for confirming/testing *a priori* hypotheses about specific ecological patterns; or (c) predictive—i.e., forecasting the effects of ecological change through time and/or space [15,16]? What are the statistical and biological populations of interest? Articulating these goals and answering these questions in advance will help interested parties to assess whether the expected data will be suitable for their intended purpose. If the aim is to improve conservation or management goals, the plan needs to explain how the data from the proposed study will contribute to those improvements. Moreover, the process of justifying research questions and goals will help all parties evaluate the merits of the proposed research, avoid potentially trivial questions/hypotheses [17] and hopefully identify and rectify potential inadequacies early. 

The research plan should address how the 3Rs—*Replacement, Reduction, Refinement* [18]—will be implemented to ensure that the proposed research is humane [19]. Within the *Replace* theme, researchers should demonstrate that alternative methods to gain the information needed are inadequate, and thus leave collaring as the only option. Analytical and technological innovations in non-invasive tools such as camera traps and fecal genetics are increasingly capable of at least partially addressing questions that previously required collar-based location data [20]. In many instances, existing literature and research (possibly relying on analogous taxa) may be sufficient for project needs. The research plan should summarize the weight of evidence that unequivocally shows that no other suitable, less invasive methods are available, with explanations as needed and a review of relevant literature.

*Reduction* of the number of individuals required is rarely a salient concern in snow leopard telemetry studies because of the inherent challenges of capturing snow leopards. It is nonetheless important to consider that once the desired sample size is achieved, captures should be stopped until a new study plan is developed that justifies the need for additional captures. Reduction can also be obtained by ensuring that all captured animals are comprehensively studied, for example, by collecting samples for future disease surveys or genetic analyses, without compromising animal welfare. Similarly, reduction may entail archiving telemetry data in a repository like Movebank [21], with an embargo period if needed (for sensitive data) and/or commitment to make data available to others upon reasonable request. This would help reduce the need for additional animals to be captured for future studies. Reduction also refers to the duration of time that the animals are affected, for example by ensuring that tracking devices are removed from the animals once the batteries are depleted. Re-capturing individuals to change collars when batteries are depleted allows the same individuals to be monitored for a long time, generating valuable data on life history, intra-specific space use dynamics, and longer-term habitat or behavioral changes. This can be justified if the aims require such data but the impact on the animals from re-captures and wearing tracking devices for a longer duration need to be considered. Non-target species that are affected should be considered as well, and reduction can be obtained by choosing a capture technique that minimizes the risk of capturing non-target animals. While reduction is an important principle, it is equally important not to use too few individuals—insufficient sample sizes may invalidate statistical approaches, particularly for inferential studies, and thus lead to inconclusive or questionable project outcomes. 

*Refine* involves improvements to minimize the negative effects on the animals and to increase the scientific benefits. It includes most aspects of the study design, from the planning of the study until the tracking device is removed from the animals. The research plan should specify what protocols will be employed to continuously evaluate and improve capture and handling procedures, how unexpected incidents will be evaluated and reported, as well as a description of when and how humane endpoints will be implemented if problems occur [11,22]. By combining telemetry studies with other methodologies such as camera-trapping or prey abundance estimation further insights can be gained compared to conducting the telemetry study in isolation. All personnel involved in captures need to be trained, or at least be familiar with the 3Rs and how to implement them in capture-related work [19].

## 3. Design

Given the logistical challenges of capturing elusive, low-density carnivores, many studies attempt to capture and collar as many animals as possible. However, this approach ignores the principle of reduction, puts animals at higher risk levels than necessary, and jeopardizes limited resources [14]. For example, empirical and mathematical evidence suggest that, with respect to resource selection (a) common subject for telemetry studies; [23]), reasonably precise and accurate estimates of population-level parameters may be generated from far fewer individuals than is typically assumed, particularly if resource selection is strong and landscapes are complex [24], as is likely the case for snow leopards across much of their distribution. Nonetheless, a standard baseline of scientific rigor in research will require that any valid collar-based study deploys collars on multiple individuals. 

If the data are intended for inference (i.e., to test clear *a priori* hypotheses), statistical power analyses drawing on pilot data or data from the literature (including analogous species where appropriate) should be conducted to determine the sample size needed to evaluate the proposed research hypotheses [1]. A pilot study may be needed to obtain the information—population-level variation in the target parameters [25]—necessary to conduct power analyses. Except for idiosyncratic and extraordinary circumstances where the objective is to describe or monitor the behavior of a single individual, even a pilot study will require that at least two individuals are monitored. Researchers interested in resource selection can use the closed-form expressions of Street et al. [24] to estimate how many animals and location fixes are necessary to detect expected selection patterns. Given the substantial effort required for capturing individuals of most felid species, it is reasonable to expect that inference at ecological scales of interest to research and conservation will require a multi-year effort to acquire sufficient sample sizes. If the primary goals involve prediction and forecasting, sampling design should reflect additional effort to ensure that there are sufficient out-of-sample data to validate predictive performance [16]. In many cases, it may be difficult to successfully achieve the target sample size, therefore the research plan should also explain and justify a minimum sample size. If estimated sample size requirements are not achievable with available resources, the study goals should be modified or refined, or delayed until additional resources can be secured. By contrast, nearly any sample size *might* be justifiable in an exploratory or descriptive context provided that the inherent limitations of such studies are recognized and communicated to stakeholders. 

Telemetry studies of solitary large carnivores (and indeed individual-based studies of free-ranging wildlife more broadly) are inevitably constrained by happenstance. Unforeseen events and equipment failures mean that even fundamental sampling design considerations, such as the number of individuals or the location fix rates, are only partially controllable. A proportion of the deployed collars will not yield the expected amount of data due to collar malfunctions, or because the animal dies or disperses outside of the study area. Accounting for this in advance means researchers should increase the target sample size by a buffer of at least 20% [26]. Further, if age or sex-specific estimates are desired (or if differences among such classes would otherwise confound inference), the sample size needs to be increased even further because the actual animals captured will inevitably not align with needs as they are likely unevenly dispersed among demographic groups. 

With the study goals and minimum sampling needs identified, the next step is to determine which tracking devices are most suitable. There are several brands that manufacture tracking devices, even though they may appear similar the quality, price, and features may differ [26]. General recommendations for suitable brands are not possible because the technology and devices develop constantly. Instead, a useful step is to consult potential vendors and researchers who have worked in similar conditions/with similar species. Performance of tracking devices of poorer quality may degrade over time, especially when deployed in field settings, for ethical reasons the best available tracking device should be selected. Vendors are often willing to work with researchers to build customized equipment if needed. Most studies suffer from malfunctioning tracking devices, in cases, these are so frequent that research goals cannot be met [27]. Notifying the vendors of malfunctions and if possible returning the tracking devices for inspections will help improve their reliability. Most biologists would prefer collars that weigh less, are more reliable/less prone to malfunctions, generate more data and cost less. Successful vendors are constantly improving their products with these factors in mind, the ones who do not succeed will go out of business. Selected tracking devices should be tested under a range of likely environmental conditions, preferably in the study area, before deployment to confirm that they operate as expected. Such tests can be performed by fitting the tracking device to a captive animal to investigate how the animal reacts to the device and by deploying the device on a domestic animal in the study area to test how well communication performs. 

Two universal variables important for every study using collars are battery life and acquisition rates of GPS positions. The latter represents the raw telemetry data and are limited by the former. The values for these are generally lower in field settings than in controlled tests, therefore, specifications provided by the vendor should be viewed as the best-case scenario [26,28]. Battery lifetime is negatively correlated to positioning and uplink interval. The appropriate positioning interval will depend on the research questions the study aims to answer: for coarse space use and life-history-related questions, one position a day and a long battery lifetime may be ideal whereas for more detailed descriptions of behavior, such as foraging strategies, an interval 30 min or less between fixes could be appropriate. Autocorrelation among fixes at short intervals no longer poses the challenge it once did to telemetry studies focused on spatial ecology, as a new generation of analytical tools has been developed [29]. Whatever fix rate is chosen, research goals and plans should reflect realistic (i.e., pessimistic) expectations of collar performance and capabilities. 

Once the target sampling parameters are determined, the collaring group must demonstrate the commitment of resources (funds, time, and personnel) to achieve this target and have success on the project. Estimates of the field effort required to obtain sufficient data are needed; evaluating similar studies can allow the estimation of how much time and human resources would be needed to capture and follow the required number of individuals and collect associated ground-based data, such as habitat descriptions of foraging studies. Capital costs for these projects relate to equipment (tracking devices, capture equipment, drugs), salaries, transportation, and fees for transmitting the data from the collars. Further, the post-capture data management and analytical needs can be considerable, and costs for a qualified scientist to manage these should be considered. A budget showing estimated costs is essential in the plan. The study should not be initiated until sufficient funding is secured. Pilot studies not only help inform sample size needs, they provide a valuable opportunity to refine logistical procedures and protocols, and to better estimate the project resource needs [25]. Pilot studies should be designed so that the data collected can be used to answer a smaller research question; in cases where this is not successful, the data should be made freely accessible or used in collaboration with other research groups, thereby increasing the sample size. 

Similarly, political support should be regarded as a finite resource and evaluated carefully; if support from permitting agencies or other governmental bodies is uncertain or tenuous, it may be difficult to achieve needed sample sizes and/or subsequent fieldwork. Snow leopard collaring often attracts public interest and media attention that can lead to additional scrutiny of researchers, organizations, and agencies engaged in collaring work [13]. In practice, relevant governmental bodies should fully understand and support the intended scale and scope of the project, the risks involved, and the plans to mitigate that risk well before capture work begins. 

**Figure 2:**
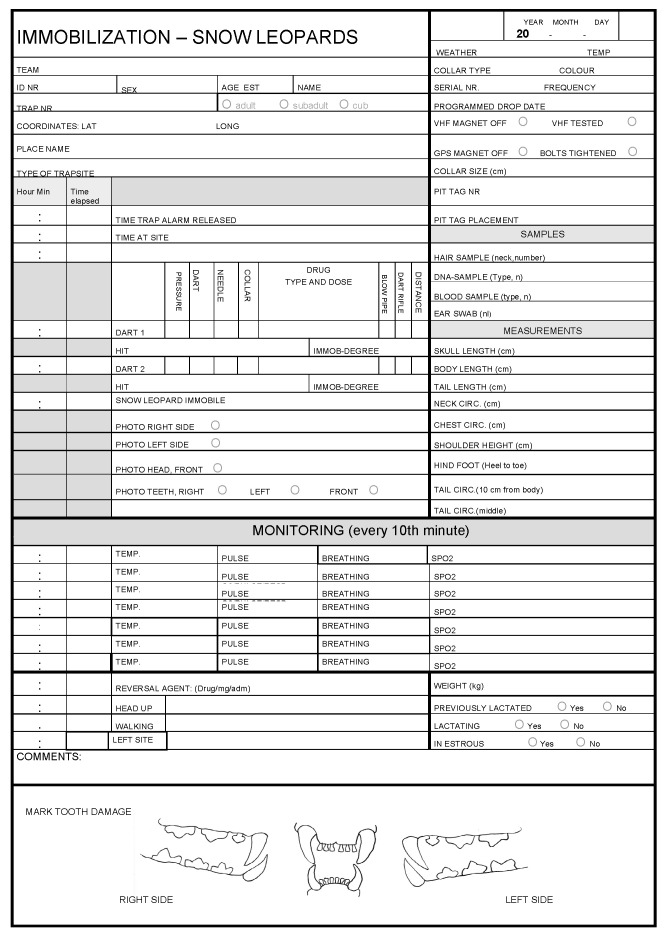
Example of a capture form used to ensure that all data is recorded and monitoring conducted at the intervals specified in the research plan.

The research plan should include a detailed itinerary of the capture process. This starts with the capture team. Captures and collaring should only be carried out by a team of professionals with proper training, experience, and expertise in wildlife capture, veterinary anesthesia and monitoring, animal handling, and basic first aid techniques to ensure that negative effects on animals are minimal, and scientific gains are maximized [22,30]. Adequate experience in managing anesthetized animals, drug dosages and delivery, and physiological monitoring are essential to quickly problem-solve the appropriate response when physiological readings deviate from normal. The plan should make clear what, and how, samples should be collected (e.g., blood, hair, buccal swabs, fecal, etc.), what measurements should be recorded, and whether the animal should be fitted with identifying objects (ear-tags, microchips). In general, prophylactic interventions (e.g., routine administration of antibiotics and antiparasitic drugs) will not be a part of standard capture protocols, as they can potentially disrupt or interfere with the ecological processes and relationships of interest. It is useful to create a data-form for the collaring process (e.g., Figure 2) to ensure that all measurements and samples are completed for each capture.

Internationally accepted standards for invasive research involving animal subjects require that studies obtain prior review and approval of any procedure involving live animals from appropriate oversight bodies and permitting agencies. Many research journals do not publish work if authors cannot show that the work meets ethical standards [31] and conforms to the legal requirements of the country in which the study occurred. For research groups with academic affiliations, approval should be sought from ethics or other similar committees. Given the inherent risks involved, teams should develop contingency and emergency protocols for first aid, euthanasia, and handling of non-target animals, as well as procedures for managing potential risks to human health (i.e., accidental drug exposures, and exposures to zoonotic diseases via contact, blood, bites, and scratches). Access to emergency medical and veterinary care is often non-existent in many of the landscapes inhabited by snow leopards. As such, protocols should be developed in direct collaboration with experienced veterinary and human medicine professionals to ensure that all proposed procedures meet acceptable thresholds for the safe and ethical treatment of the animals and that risks to capture teams are minimized. 

## 4. Implementation

A detailed description specifying capture methods, the safety measures that will be employed, and the qualifications and experience of the staff should be included in the study plan. The plan should specify an assessment of the potential risks for both animals and staff and outline the procedures to follow to mitigate risks when they are presented. It is advised to review all possible capture methods and seek advice from multiple, experienced parties on which methods are best suited for the proposed study area [32]. 

Several different heuristics for collar size and weight limits have been employed, e.g., the collar weight should not exceed 2, 3, or 5% of the animals’ weight [30,33,34]. Wilson et al. [35] showed that when animals moved at a high speed, forces exerted by the collars increased dramatically: a collar weighing 3% of a Cheetah’s (*Acinonyx jubatus*) body weight exerted forces equivalent to 54% of the body weight. Care should be taken to select a collar model as light as possible; for felids that are dependent on agility and speed, collars should probably weigh less than 2% of the animals’ body weight. It is necessary to select a collar model that can be adjusted to fit the animal, thereby avoiding ulcerations [1,36]. If capturing a young or thin animal, the collar should be fitted so that the animal has room to grow e.g., [37]. Care must be taken to ensure that the collar is not fitted so loose that it can get wedged in the animal’s mouth or that a front leg or object can get caught [11]. If the information on sex-specific neck sizes is available for the target species, these should be used as minimum sizes for young individuals. 

Collars should preferably be fitted with a programmable mechanism (drop-off) that detaches the collar from the animal at a time decided by the research team [38], or at a minimum a break-away zone consisting of a material that will degrade over time (e.g., cotton [39]) ensuring that the collar eventually detaches. Realtime locations can expose the animals to poaching risks or stress from tour operators, especially for charismatic and elusive species. It is therefore critical that real-time locations are not shared publicly and that VHF-frequencies and GPS-collar data are only accessible by personnel that require the information in their work. 

## 5. Snow Leopard Captures Using Foot Snares

For many large felids, including snow leopards, the most widely implemented capture method is foot snaring [40,41]. Snares require training and experience to be set safely and effectively [42]. Here we provide an overview of some important considerations, but it is not a substitute for training and experience. The following points should be considered repeatedly during study design and implementation when using snares:

### 5.1. Site Selection

Leg-hold traps such as snares are relatively easy to transport and carry, even in rugged landscapes, allowing the researchers to carefully choose the terrain where the animal will be sedated and later safely recover. In addition to selecting sites that snow leopards are likely to visit, it is imperative that trap sites are selected so that undue injuries are avoided. The following safety considerations must be evaluated specifically for snow leopard captures, but are also relevant to other felid captures:

1. A snared snow leopard will thrash around across the area it can reach. Therefore, it is imperative that the total length of the snare and anchor cable be as short as possible to minimize the cat’s range of mobility, exposure to rocks or other sharp objects, and the possibility of entanglement. Although no sites in the mountains are completely safe, it is important to avoid places with dangerous features that may injure the animal and areas with ledges or cliffs where an exhausted cat could fall and be left hanging. Snares should be located away from streams or other open water sources as snared and anesthetized animals can drown even in shallow water, and wet animals are more prone to hypothermia or other physiological challenges.
2. A safe snare must be secured to an anchor that is sturdy enough to hold any animal, wild or domestic (e.g., bears or camels), that might step into it—anything less creates the risk of debilitating injuries to the animal and research staff alike.
3. Sites must be considered both for their probability of capturing snow leopards, as well as for effectively approaching and darting captured animals.
4. Sites should be selected to maximize the likelihood of snow leopard captures, but also minimize the risk of capturing non-target species. Sites used regularly by non-target species should be avoided. Similarly, baits and lures should typically not be used as they will likely attract scavenging species to the snare, increasing captures of non-target species. Snares may be particularly dangerous to certain non-target species, especially ungulates, which can respond more violently to capture, increasing the risk of debilitating or life-threatening injuries.
5. When a snow leopard recovers from chemical immobilization, it tries to move into steep, rugged terrain as that is their natural escape response. To avoid injuries from falls before the animal has fully recovered, it is important to ensure that there are gentle slopes in at least one direction into which the animal may be guided if necessary. If there is no such place, it is important to identify a site to which the animal can be carried after it has been darted, or alternatively, it is allowed to recover in a secured, on-site, enclosure for later release. If an animal has been critically injured and will not be able to recover it is ethically justifiable to euthanize it, the capture team should be trained and equipped to carry out humane euthanasia [12].
6. Researchers need to be able to safely access the site under a wide range of conditions, including inclement weather and darkness. Snares must be avoided in sites that may become inaccessible under adverse conditions (e.g., crossing of flooded waterways).

### 5.2. Monitoring of Traps

The cumulative risks of injuries and mortality increase with the time that the animal stays caught in the trap—for some species/individuals the risk may be constant per unit time whereas others may become more desperate to break free the longer they have been trapped [31]. This is perhaps even more critical when using traps such as snares that do not confine the animal in a small space [22]. There are several different types of trap surveillance solutions, utilizing radio, cell phone, or satellite communication. Cell phone (GSM) technology is relatively cheap and easy to use. Trap transmitters are programmed to send text messages to cell phones and/or email addresses with status updates and notifications if the trap is triggered. Cell phone systems are dependent on a reliable GSM network. In areas without GSM coverage, one can use satellite communication-based trap transmitters. For such a system to work it is dependent on internet access in the living quarters of the capture team. In areas that lack a GSM network or internet access, the traps can be equipped with VHF-transmitters. To ensure that animals are not trapped for longer periods than necessary, these transmitters should be monitored with custom-made systems that provide constant trap monitoring, e.g., [43]. Using such a system, the average time from capture to darting of 22 snow leopards was 28 min (range 13–48 min). If the signal from a transmitter indicates that a trap is triggered, the capture team should immediately mobilize as per a rehearsed and practiced tactical plan, with all necessary equipment at hand (which necessitates that all equipment and capture team members be at the ready at all times). The same protocols apply if the signal from the trap transmitter cannot be heard, as a captured animal may have displaced the transmitter or the antenna. A transmitter that cannot be monitored should be treated as a triggered trap and responded to appropriately.

Even with trap monitoring systems in place, traps must also be visited and inspected as often as possible, preferably once every day to ensure they remain in working order, have not been disturbed, and that no animal has been caught without triggering the alarm. 

### 5.3. Drug Delivery System 

Snow leopards can be darted using blow pipes or CO_2_-powered dart rifles. Blow pipes propel the darts with little force, and the risk of injury due to poor dart placement is low especially if aiming at the thigh. Delivering darts accurately with a blow pipe, however, is difficult when the distance exceeds 5 m. At the high altitudes where snow leopards occur, capture personnel may not be able to propel the darts with the same force as at lower elevations, further reducing the effective range for blow pipes. Gas-propelled dart rifles can shoot darts accurately at greater distances. However, injuries can occur if pressure is set too high or dart placement is poor. Weather conditions, especially heavy wind, will also affect the ballistics and accuracy of a dart. Snow leopards tend to lie down when the capture personnel are 10–20 m away, whereas when approached closer they react and commonly face the crew. To allow a safe dart placement, it may be necessary for a member of the capture team to walk away from the darter and draw the attention of the snow leopard so that the animal presents a clear shot at the thigh for the darter. Induction times can be considerably longer in stressed and aroused animals [12]; all team members must leave the site immediately after the darter has assessed that the dart has injected the drugs to allow a quick absorption of the drugs. 

### 5.4. Handling of Animals

The purpose of the immobilization is almost always to fit a collar and collect samples under safe and ethically approved conditions. Rarely is it necessary to achieve complete anaesthesia (unconsciousness), and, consequently, the type of drug and dosage used is selected to allow for a rapid recovery and minimal side effects such as respiratory or cardiac depression. The most commonly used drugs to immobilize free-ranging snow leopards have been a combination of tranquilizers/sedatives and anesthetics, e.g., medetomidine and ketamine or medetomidine and zolazepam-tiletamine [41,44]. Because the snow leopards are not completely anaesthetized they will feel pain and can react to outside stimuli such as sounds and light. Staff attending captures should speak with low voices and treat the animals gently and with care to avoid stressing them, or in the worst case temporarily arousing them. Captures should only be conducted by professional staff with adequate training and experience to conduct the work in a safe and ethical way. A correctly calculated drug dose, allowing rapid recovery and sufficient anaesthesia, induces immobilization for 40–60 min on average. Completing the entire operation within that time frame requires a plan with designated tasks and that the team works efficiently. A blindfold should be applied to the snow leopard to prevent debris from getting caught in the eyes, damaging the eyes with strong headlamps or sunlight, and to avoid visual stimuli that can stress the animal. 

Immobilized animals should be monitored throughout the capture until they recover. Properly planned and executed monitoring allows for rapid recognition of expected and unexpected complications. Consistent monitoring and recording (See Figure 2) throughout the chemical immobilization event enable the capture team to prevent or intervene ahead of those complications through real-time analysis of trends. Should any parameter deviate outside the normal range or show a trend that is considered not to be normal it is necessary that staff have knowledge and ability of how to respond. Once immobilized, the animal should be placed into a position that allows accurate and effective monitoring and does not affect breathing or stops circulation. The most important parameters to monitor are airway, rectal temperature, depth of anesthesia, respiration (rate and depth) and cardiovascular function (heart rate), and the related oxygen saturation in the blood (as measured by pulse oximetry). Snow leopards appear to cool down rapidly if exposed to wind, even at relatively mild temperatures around 0 °C (Johansson. Unpublished data). Similarly, exposure to direct sunlight can cause hyperthermia. All observations, samples, measurements, and activities undertaken during the immobilization should be recorded on a data form. Video documentation should also be recorded if possible, as it may be very helpful to review if issues arise during the capture process. In addition, given that the opportunity to take measurements and samples from free-ranging snow leopards is rare, every effort should be made to collect as much data as possible. 

### 5.5. Recovery

The cat should be allowed to recover in a well-shaded place if the ambient temperature is warm. The animal should be covered with a blanket or sleeping bag if it is windy or colder than −10 °C. Attention must be paid to the wind as wind-chill can reduce body temperature rapidly, even at mild temperatures. The animal should be positioned so that it can breathe easily: snow leopards should be positioned on either side on as flat ground as possible, the neck kept straight and the nose clear. If the ground is uneven, the head should be higher than the body, while the nose should not point upwards to avoid the aspiration of fluids. A soft cloth or towel can be placed under the head and the blindfold left loosely over the eyes. The animal must recover at its own pace as it metabolizes the drug. Most, if not all, sedatives used for snow leopards can be reversed with an antagonist whereas there are no antagonists for the tranquilizers, see [12] for a detailed list of drugs used to immobilize snow leopards. The antagonist should not be given until most of the tranquilizer has been metabolized to allow for a smooth recovery. Care must be taken to avoid premature recovery where the animal moves away before it is fully awake and coordinated. Such an animal can cause injury to itself and/or to those present. Capture personnel must move away from the animal and observe it from a distance until it leaves the site. At nighttime, flashlights can stress the animal, so it is important to wait until the animal starts to move, after which the personnel should leave the site, and return after an hour to check. VHF receiver can be used to confirm that the animal has left the site. Any nearby snares must be disarmed or covered to avoid recapturing the recovering animal. It may be necessary to leave snares closed for several days until the animal has moved away from the trap area to avoid re-capturing, and sedating it twice in a short time span. 

## 6. Conclusions

For telemetry studies to be scientifically sound and ethically justifiable, they must be founded on well-developed research plans where animal welfare is the first priority. Critically evaluating proposed aims in relation to expected outcomes increases the chances that the data collected can answer the questions of interest. With careful planning and an experienced capture team, it is possible to employ foot snares to safely capture snow leopards in their remote mountain habitat. Such studies on snow leopards and other low-density, rare species can prove essential for filling knowledge gaps relevant for enhancing or improving conservation outcomes.

## Data Availability

Not applicable.
